# Transcriptomic Signatures of Experimental Alkaloid Consumption in a Poison Frog

**DOI:** 10.3390/genes10100733

**Published:** 2019-09-21

**Authors:** Eugenia Sanchez, Ariel Rodríguez, Jose H. Grau, Stefan Lötters, Sven Künzel, Ralph A. Saporito, Eva Ringler, Stefan Schulz, Katharina C. Wollenberg Valero, Miguel Vences

**Affiliations:** 1 Zoological Institute, Technische Universität Braunschweig, 38106 Braunschweig, Germany; m.vences@tu-bs.de; 2 Department of Biology, Stanford University, Stanford, CA 94305, USA; 3 Institut fur Zoologie, Tierärztliche Hochschule Hannover, 30559 Hannover, Germany; Ariel.Rodriguez@tiho-hannover.de; 4 Museum für Naturkunde Berlin, Leibniz-Institut für Evolutions- und Biodiversitätsforschung, 10115 Berlin, Germany; jh.grau.jipoulou@gmail.com; 5 Biogeography Department, Trier University, 54296 Trier, Germany; loetters@uni-trier.de; 6 Department of Evolutionary Genetics, Max Planck Institute for Evolutionary Biology, 24306 Plön, Germany; kuenzel@evolbio.mpg.de; 7 Department of Biology, John Carroll University, University Heights, OH 44118, USA; rsaporito@jcu.edu; 8 Messerli Research Institute, University of Veterinary Medicine Vienna, Medical University of Vienna, and University of Vienna, A-1210 Vienna, Austria; eva.ringler@univie.ac.at; 9 Department of Integrative Zoology, University of Vienna, A-1090 Vienna, Austria; 10 Institute of Organic Chemistry, Technische Universität Braunschweig, 38106 Braunschweig, Germany; stefan.schulz@tu-bs.de; 11 Department of Biological and Marine Sciences, University of Hull, Kingston-Upon Hull 01482, UK; kc.wollenberg@gmail.com

**Keywords:** Dendrobatidae, Aromobatidae, *Dendrobates*, Allobates, epibatidine, gene expression, feeding experiment, immune system, resistance

## Abstract

In the anuran family Dendrobatidae, aposematic species obtain their toxic or unpalatable alkaloids from dietary sources, a process known as sequestering. To understand how toxicity evolved in this family, it is paramount to elucidate the pathways of alkaloid processing (absorption, metabolism, and sequestering). Here, we used an exploratory skin gene expression experiment in which captive-bred dendrobatids were fed alkaloids. Most of these experiments were performed with *Dendrobates tinctorius*, but some trials were performed with *D. auratus*, *D. leucomelas* and *Allobates femoralis* to explore whether other dendrobatids would show similar patterns of gene expression. We found a consistent pattern of up-regulation of genes related to muscle and mitochondrial processes, probably due to the lack of mutations related to alkaloid resistance in these species. Considering conserved pathways of drug metabolism in vertebrates, we hypothesize alkaloid degradation is a physiological mechanism of resistance, which was evidenced by a strong upregulation of the immune system in *D. tinctorius*, and of complement C2 across the four species sampled. Probably related to this strong immune response, we found several skin keratins downregulated, which might be linked to a reduction of the cornified layer of the epidermis. Although not conclusive, our results offer candidate genes and testable hypotheses to elucidate alkaloid processing in poison frogs.

## 1. Introduction

Alkaloids are unpalatable or toxic substances, and have been documented in five families of anurans [1,2,3,4,5]. In one of these families, Dendrobatidae, the ability to uptake and store alkaloids from the diet, known as sequestering, evolved independently at least four times [6,7,8]. In this family, sequestering has been shown as the main predictor of aposematism [8,9], which is the association of unprofitability with a warning signal to predators, usually a conspicuous coloration. However, aposematism in dendrobatids is considered a complex phenotype because it has been associated with many other traits, such as diet specialization, high active metabolic rate, high rate of molecular evolution and speciation, high calling rates at lower frequencies, conspicuous behavior, and larger body mass and body size [6,8,10,11,12,13,14,15,16,17,18]. Hence, to understand the complex evolution of aposematism in this family, it is paramount to investigate the molecular basis of alkaloid processing.

The processing of alkaloids in dendrobatids includes their absorption, metabolism and sequestering. Alkaloid absorption might occur along the digestive system through simple diffusion, given their lipophilic nature [19] or through ABC transporter proteins [20]. Once absorbed, alkaloids might either circulate freely, as part of lipoproteins, or bind to carrier protein(s). However, before they reach the tissue of storage, they could (or not) be metabolized. For example, they could be biotransformed by cytochrome P450 enzymes (hereafter CYPs) in the liver [20], which have been found differentially expressed in a study comparing wild and captive *Oophaga sylvatica* [21]. However, from a biochemical point of view, alkaloid processing in poison frogs is remarkable because they are sequestered without modifying their molecular structure in most cases [5]. The binding of alkaloids to carrier protein(s) might protect them from biotransformation, and recently a potential carrier protein, saxiphilin, has been identified in a dendrobatid [21]. Finally, either freely or bound to a carrier, alkaloids should be internalized by storage tissues. Although alkaloids have been found in several tissues of poison frogs [22], they are mainly stored in the skin, purportedly in the granules of the granular glands [23,24,25]; hence, the skin is a target organ in which to study alkaloid internalization and storage.

An important part of alkaloid processing is resistance to autotoxicity. The best-studied mechanism of resistance in poison frogs is the accumulation of mutations in target ion-channels or receptors [9,26,27,28,29,30,31]. For instance, the poison frog alkaloid epibatidine [32], which we used in the present study, acts by binding to nicotinic acetylcholine receptors [33]. During the evolution of dendrobatids, nucleotide substitutions in the binding site for this receptor have been selected, which confer resistance yet leave the receptor’s function virtually unchanged [9]. However, resistance to alkaloids might also be achieved through other mechanisms. Storage of alkaloids could be considered a mechanism of resistance [34] because it involves compartmentalization, i.e., the storage of most of the alkaloids in a reduced number of organelles, cells or tissues, which limits their interaction with target tissues such as the muscles [35]. The mechanisms behind receptor modification and alkaloid storage might have evolved through selection in lineages recurrently exposed to alkaloids [32,36], possibly through diet specialization [6,7,11,37,38]. Other mechanisms to deal with autotoxicity might involve the metabolism of dietary alkaloids [34]. For example, in vertebrates, the biotransformation of small molecules usually makes them more hydrophilic, making it easier to excrete them through urine and/or allowing the binding of other molecules for their degradation [19].

In this study, we performed a series of feeding experiments coupled with gene expression analyses on skin samples from captive-bred poison frogs to gain insights into the molecular responses of poison frogs to alkaloids. Our working hypothesis was that alkaloid processing pathways would be differentially expressed in frogs with control and alkaloid diets. More specifically, we predicted that, in captive-bred alkaloid-free frogs, any alkaloid-related pathways would be expressed in basal amounts, whereas these same pathways would be overexpressed in captive-bred frogs fed with alkaloids for the first time. Furthermore, we expected that their expression over time would be regulated due to the half-life of the proteins involved in these pathways. We further hypothesized that some components of the alkaloid processing pathways are conserved among dendrobatids and, hence, different species would present similar gene expression patterns upon alkaloid consumption.


**Glossary**
Alkaloid absorptionUp-take of alkaloids from dietary items.BiotransformationStructural change of a compound. For example, the conversion of pumiliotoxin (+)-**251D** into allopumiliotoxin (+)-**267A** known from *Dendrobates* and *Adelphobates* [39].Alkaloid degradationBreakdown of alkaloids or alkaloid derivatives.Alkaloid metabolismBiotransformation, excretion or degradation of alkaloids.BioaccumulationAccumulation of alkaloids because they are not metabolized at the same rate in which they are absorbed.Alkaloid sequesteringAbility to absorb alkaloids, and internalize and store them in selected tissues or cells.Alkaloid resistanceAvoidance of alkaloid toxicity. It can be achieved through several mechanisms, including mutations in target ion-channels and receptors, sequestering or metabolism.Alkaloid processingAbsorption, metabolism and sequestering of alkaloids.

## 2. Methods

### 2.1. Poison Frogs

We performed alkaloid feeding experiments on captive-bred poison frogs of the family Dendrobatidae (here defined as containing Dendrobatinae and Aromobatinae; see discussion in [36,40,41,42]. Nine *Dendrobates tinctorius* and four *D. auratus* were obtained from Understory Enterprises (Charing Cross, Ontario, Canada) and DendroWorld (Bullecourt, France), two *D. leucomelas* and two *Epipedobates anthonyi* were obtained from Terraristik Braunschweig (Brunswick, Lower Saxony, Germany), and three *Allobates femoralis* were obtained from the frog colony at the University Vienna, Austria. Frogs were full-siblings specifically bred for our experiments (see Table SM2.1 for detailed information on the experimental subjects). Frogs were kept in independent plastic containers (17.5x11x12 cm) under similar conditions of light and temperature during the experiments. All the animals sampled were at least one year old prior to the commencement of experiments. Permission to house poison frogs was issued by the Veterinäramt Stadt Braunschweig in Germany, and feeding experiments were ethically approved by the Niedersächsisches Landesamt für Verbraucherschutz und Lebensmittelsicherheit (No. 33.19-42502-04-15/1900) in Germany. Additionally, we sampled two wild-caught *D. leucomelas* in Guri, Bolívar State, Venezuela (8°18’51’’N, 62°39’56’’W), under collection permits issued by Oficina Nacional de Diversidad Biológica (No. 0908 and No. 0217) in Venezuela.

### 2.2. Experimental Design for Gene Expression

**Figure 1:**
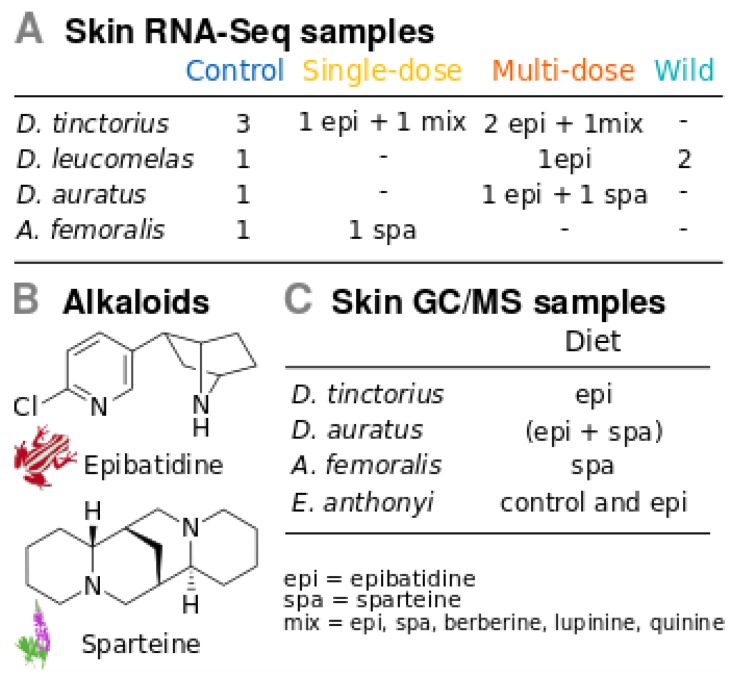
Experimental design. **A.** Sample sizes per treatment and species used for RNA-seq. **B.** Structure of the two main alkaloids used in our study: epibatidine, which occurs naturally in frogs *Epipedobates* spp., and sparteine, which occurs naturally in plants *Lupinus* spp. **C** Individuals fed long-term with alkaloids to confirm whether they sequester these alkaloids using GC/MS.

Three different experimental treatments were applied: (i) control, in which frogs were maintained on an alkaloid-free diet for eight days, (ii) single-dose treatment, in which frogs were on an alkaloid-free diet, but fed with alkaloids on the seventh day, and (iii) multi-dose treatment, in which frogs received an alkaloid diet throughout the eight days of the experiment (Figure 1A, Table SM2.1). The same amount of food was offered to each animal, 8-12 fruit-flies (*Drosophila* sp.) per day. Flies were dusted with either vitamin powder (Nekton-Rep, NEKTON GmbH, Germany) in the control, or with a mixture of vitamin powder and alkaloids for the single-dose and multi-dose treatments. We administered three different alkaloid composition treatments: epibatidine only (2% m/m), sparteine only (2% m/m), or a mixture of epibatidine, sparteine, berberine, lupinine and quinine (1% m/m each) (Figure 1A, Table SM2.1). Although we tried to control the quantity of alkaloid given to each frog by offering the same number of fruit-flies and having a mixture with a known concentration of alkaloid, it is impossible to know how much alkaloids were consumed by each frog. Epibatidine was used because it is the only poison frog alkaloid commercially available, although it has naturally been found only in *Epipedobates* and *Ameerega* spp. [32]. Because epibatidine is a highly toxic alkaloid (LD50 ~0.2 mg/kg; [43]), and *A. femoralis* is considered a non-toxic dendrobatid species ([44], but see [45] and [46]), we judged using the less toxic alkaloid sparteine (LD50 ~200 mg/kg; [47]) for this species. Sparteine is an alkaloid from plants of the genus *Lupinus*, which has a similar structure to epibatidine but belongs to the quinolizidine class (Figure 1B).

### 2.3. RNA-Seq, Transcriptome Assemblies and Annotation

We used whole skin samples because initially we wanted to focus on sequestering, especially alkaloid internalization and storage by the skin. These samples were taken 10 minutes after the last feeding, and stored in RNA-later at -80°C. RNA was extracted using a TRIzol protocol and prepared for sequencing with the Illumina TruSeq Stranded mRNA Library Prep protocol (San Diego, CA, USA). Sequencing was carried out on Illumina NextSeq sequencer (Max Planck Institute for Evolutionary Biology, Plön, Germany) using NextSeq V 500/550 High Output Kit v2 (2 × 75 bp paired-end; San Diego, CA, USA). Sequences were quality-trimmed with Trimmomatic 0.32 (slidingwindow:4:10 leading:5 trailing:5 minlen:50; [48]) and normalized with BBnorm (target = 40 mindepth = 2; https://jgi.doe.gov/data-and-tools/bbtools/). The transcriptome of each species was de-novo assembled from the quality-trimmed reads of each sample using Trinity 2.1.1 (--min_kmer_cov 2 --SS_lib_type RF; [49]). We used DRAP [50] with default parameters to maximize compactness while reducing chimerism and assembly errors in the transcriptomes. Only transcripts with FPKM > 1 (Fragments Per Kilobase of transcript per Million mapped reads) were kept for further analyses. The quality of the transcriptomes was evaluated with TransRate [51] and BUSCO v3 [52]. Transcriptomes were annotated using Annocript [53] with the default configuration, with the SwissProt and UniRef90 as reference databases (March 2017; [54]). For each sample, reads were mapped to the corresponding species transcriptome using Bowtie [55], and abundances per transcript were estimated with RSEM [56] as implemented in the Trinity protocol [49].

### 2.4. Gene Expression in Dendrobates Tinctorius

The *D. tinctorius* dataset was represented by a minimum of replicates per treatment (control n = 3, single-dose n = 2, multi-dose n = 3; Figure 1A), and was used to identify gene expression patterns associated with alkaloid processing pathways. This dataset was analyzed at the transcript level as there is no gene-transcript map available for the species and we did not want to exclude new yet unannotated genes from the analysis. Abundances were first normalized by library size with *edgeR* [57] using TMM (Trimmed Mean of M-values). Using this matrix, we built a multidimensional scaling plot with *edgeR* function *plotMDS*, and performed an analysis of similarities (ANOSIM) between treatments using the package *vegan* [58]. Differential expression analyses were performed in *edgeR* for the comparisons single-dose vs control, multi-dose vs control, single-dose vs multi-dose, and alkaloid (i.e., single-dose and multi-dose together) vs control, with the cut-offs false discovery rate (FDR) < 0.05 and absolute log-fold change (logFC) > 2. Based on the transcript annotation, genes that contained both up- and down-regulated transcripts were not used for downstream analyses.

To test if the differentially expressed genes were functionally connected (i.e., not random), we generated interaction networks using the STRING database [59], as implemented in the StringApp plug-in for Cytoscape 3.7.0 [60]. For this, we used the best blast hits of *D. tinctorius* predicted proteins on the human proteome. Briefly, StringApp builds a network of the input genes, and compares its connectivity (number of interactions) to the connectivity of other networks of similar size generated with random sets of genes.

To test whether Gene Ontology (GO) terms were enriched in the differentially expressed transcripts, enrichment analyses were conducted with the StringApp using the human GO dataset as a reference and a cutoff value of FDR < 0.05.

### 2.5. Multi-Species Comparison

The multi-species dataset contained data of experimentally fed *D. tinctorius*, *D. leucomelas*, *D. auratus*, and *A. femoralis*, and wild *D. leucomelas* (Figure 1A). For the transcriptome of each species, we generated a SwissProt annotation – transcript mapping file that was used to summarize the estimated abundances by gene (SwissProt annotation) with the R package *tximport* [61]. These abundances were further normalized across species by library size using TMM in *edgeR*. This matrix was used to calculate logFC between treatment-control pairs using the R package *gtools*. It contained 11 treatment-control pairs, including single-dose, multi-dose and wild individuals versus their controls. Controls were control individuals of the same full-sibling group (see SM1), or, in the case of wild *D. leucomelas*, the control was the control captive-bred individual.

Multi-species data were explored using two different approaches. First, we specifically examined the genes found to be differentially expressed in the *D. tinctorius* comparisons single-dose vs control, multi-dose vs control, and alkaloid vs control. Second, we searched for genes with similar strong expression patterns (logFC > 2 or logFC <-2) in at least 6 out of 11 treatment-control pairs. The expression patterns of these genes were visualized across species using heatmaps.

Alignments of transcript sequences with positive blast hits on the nicotinic acetylcholine receptor ß2 (CHRNB2) were used to explore whether the species used in this study have mutations conferring resistance, according to [9] because it has not been previously reported for the *Dendrobates* species here included. CHRNB2 is one of the main targets of epibatidine [33], which was the alkaloid we used for feeding experiments in *D. tinctorius*, *D. leucomelas*, and *D. auratus*.

### 2.6. Alkaloid Sequestering Confirmation Tests

To test whether the two supplied alkaloids, epibatidine and sparteine, were effectively sequestered by poison frogs, we conducted long-term (min. 1 month) feeding experiments on a different set of frogs (Figure 1C). Captive bred frogs were fed with fruit flies dusted with a mixture of vitamin powder and alkaloid at 2% m/m. For *E. anthonyi,* one individual was fed with alkaloid-free flies as a control and another individual was fed with epibatidine dusted flies; for *D. tinctorius,* one individual was fed with epibatidine; for *D. auratus*, one individual was fed with a mixture of sparteine and epibatidine; and for *A. femoralis*, one individual was fed with sparteine (Figure 1C; SM2). One wild *D. leucomelas* was used for comparative alkaloid profiling (i.e., as natural positive reference). Frogs were rinsed with milli-Q water to avoid external contamination, and whole skin samples were taken and placed in approximately 400 μL dichloromethane. Samples were stored at −20°C until processing. For gas chromatography-mass spectrometry (GC / MS) analysis, 1 μL of the extract was injected into an Agilent 7890A GC system (Technische Universität Braunschweig, Braunschweig, Germany) containing a HP-5MS-fused silica capillary column (30 m, 0.25 mm i.d., 0.25 μm flm) with an Agilent 5975C inert mass detector. Analyses were carried out using an inlet pressure of 77.1 kPa with helium flow of 1.3 ml /min. The transfer line was heated to 300°C and the electron energy set to 70 eV. GC program was as follows: 5 min at 50°C, and then a temperature gradient of 10°C per minute until 320°C. Injections were performed in split-less mode with 60 s valve time.

## 3. Results

### 3.1. Transcriptomes

The assembled transcriptomes of dendrobatids (SM2) contained between 50 and 85 thousand transcripts each, had a Transrate assembly score of 0.15-0.21, and recovered about 76-89% of the BUSCO ortholog groups for vertebrates. Between 36 and 49% of the transcripts of each transcriptome were annotated to the SwissProt or UniRef90 databases.

### 3.2. Gene Expression of D. Tinctorius

**Figure 2:**
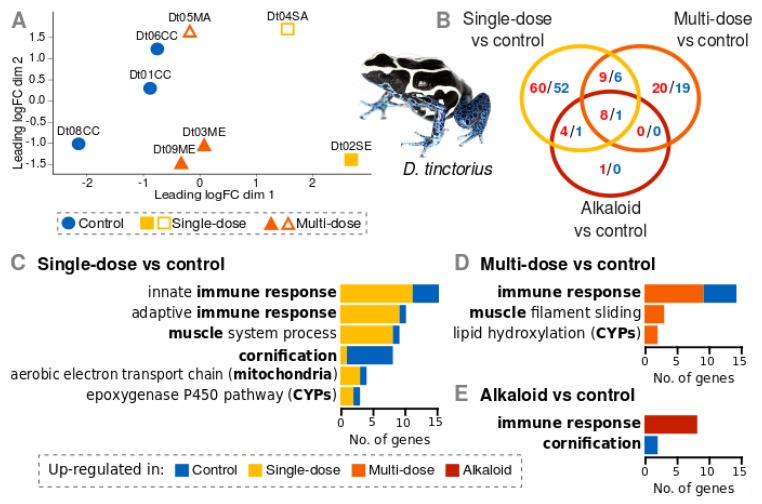
Gene expression of *D. tinctorius* upon a single dose or multiple doses of alkaloids. **A.** MDS plot showing the expression distances between samples coded by treatment. Distances were calculated from the logFC of the 500 transcripts with the highest standard deviations between conditions. Closed symbols = epibatidine. Open symbols = alkaloid mixture. **B.** Venn diagram showing the number of differentially expressed genes found for the three comparisons single-dose vs. control, multi-dose vs. control, and alkaloid (i.e., single-dose and multi-dose) vs. control. Numbers in red indicate over-expression in the alkaloid treatment, and numbers in blue indicate over-expression in the control. **C.** Enriched biological process GO-terms for the single-dose vs control comparison **D.** Enriched biological process GO-terms for the multi-dose vs. control comparison. **E.** Enriched biological process GO-terms for the alkaloid vs. control comparison.

In *D. tinctorius*, gene expression differed among treatments (ANOSIM R=0.4, *p* = 0.029). In the MDS plot (Figure 2A), samples clustered by treatment, in which multi-dose individuals were closer to the control ones than the single-dose individuals (x-axis). There was also a separation between individuals that consumed only epibatidine, and those that consumed the alkaloid mixture (y-axis), although this factor is confounded with the genetic full-sibling groups (SM2).

For the single-dose vs control comparison, 313 transcripts were differentially expressed (186 down- and 127 up-regulated). Following their SwissProt annotation, these transcripts correspond to 141 genes (81 up- and 60 down-regulated; Table SM3.1). The number of interactions among differentially expressed genes was significantly higher than expected from random sets of genes (*p*-value = 1.0e-16; Figure SM4.1). Non-redundant enriched biological process GO-terms (Figure 2C) included innate immune response (GO:00045087, FDR = 0.045), adaptive immune response (GO:0002250, FDR = 0.023), muscle system process (GO:0003012, FDR = 0.049), cornification (GO:0070268, FDR = 0.009), aerobic electron transport chain (GO:0019646, FDR = 0.049; hereafter respiration) and epoxygenase P450 pathway (GO:0019373, FDR = 0.037; all CYPs).

For the multi-dose vs control comparison, 112 transcripts were differentially expressed (49 down- and 63 up-regulated). These were annotated to 63 genes (37 up- and 26 down-regulated; Table SM3.2). The number of interactions among these genes was significantly higher than for random sets of genes (*p*-value = 1.35e-6; Figure SM4.2). Non-redundant enriched biological process GO-terms (Figure 2D) included immune response (GO:0006955, FDR = 0.030), muscle filament sliding (GO:0030049, FDR = 0.030) and lipid hydroxylation (GO:0002933, FDR = 0.030; all CYPs).

For the single-dose vs multi-dose comparison, 42 transcripts were differentially expressed (16 over-expressed in the single-dose and 26 in the multi-dose), corresponding to 27 genes (11 over-expressed in the single-dose and 16 in the multi-dose; Table SM3.3). The number of interactions among these genes was not significant when compared to a random set (*p*-value = 0.259; Figure SM4.3), and biological process GO-terms were not enriched. It is, however, worth mentioning that the mitochondrial gene for CO1, previously related to the GO-term aerobic electron transport chain, was over-expressed in single-dose when compared to the multi-dose treatment.

For the alkaloid vs control comparisons, 24 transcripts were differentially expressed (17 up- and 7 down-regulated), corresponding to 15 genes (13 up- and 2-regulated; Table SM3.4). The number of interactions among these genes was significant when compared to a random set (*p*-value = 0.0002; Figure SM4.4). Most up-regulated genes were related to the GO-terms (Figure 2E) immune response (GO:0006955, FDR = 6.06e-05), and the two down-regulated genes were keratins related to the GO-term cornification (GO:0070268, FDR = 0.0351).

There was an overlap of 24 genes (17 up- and 7 down-regulated) identified in the single-dose vs control, and multi-dose vs control comparisons (Figure 2B). Of these genes, 9 overlapped with the alkaloid vs control comparison (8 up- and 1 down-regulated; Figure 2B). Up-regulated genes were related to immune response, and the downregulated gene was a keratin.

See SM2 for all GO-enrichment results, including Cellular Component and Molecular Function.

### 3.3. Multi-Species Comparison

**Figure 3:**
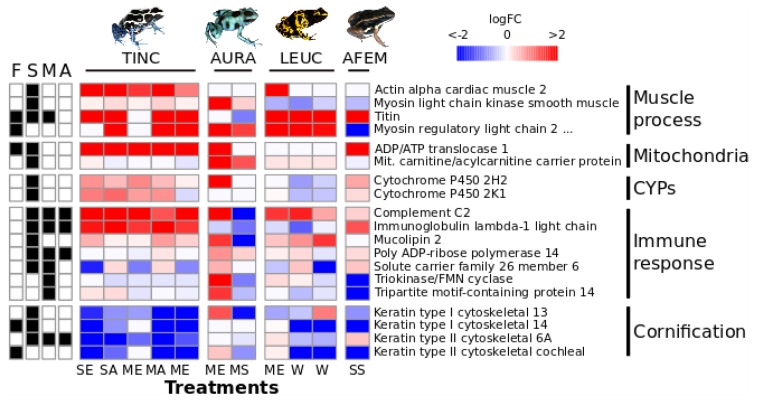
LogFC of genes of interest found across species. Species on the top: TINC = *D. tinctorius*, AURA = *D. auratus*, LEUC = *D. leucomelas,* AFEM = *A. femoralis.* Treatments on the bottom: SE = single-dose epibatidine, SA = single dose alkaloid mixtures, ME = multi-dose epibatidine, MA = multi-dose alkaloid mixture, MS = multi-dose sparteine, W = wild. On the left, we indicated, with a box filled black, the approaches used to identify each gene. Approaches: F = filtering by expression pattern, S = single-dose vs control results for *D. tinctorius*, M = multi-dose vs control results for *D. tinctorius,* A = alkaloid (i.e., single-dose and multi-dose) vs control results for *D. tinctorius*. On the right, genes have been grouped according to their broad biological significance. For visualization, this heatmap has a ceiling of logFC ± 2. LogFC values are given in SM5.

The final multi-species dataset contained 3839 genes, including 62 of the genes identified as differentially expressed in the single-dose vs control and multi-dose vs control comparisons for *D. tinctorius* (Figure SM6.1, and Table SM5.1). After filtering by logFC > ±2 in 6/11 treatment pairs, we identified 27 genes with similar patterns of expression across species (Figure SM6.2, Table SM5.2). Some of the genes identified using both approaches could be roughly related to muscle process, mitochondria, CYPs, immune response, and cornification, according to their associated biological process GO-terms (Figure 3).

When comparing the sequences of CHRNB2 of these poison frogs, none of the species tested had the known mutations conferring resistance to epibatidine (Table SM2), in agreement with previous data on other species of the same major dendrobatid clades [9].

### 3.4. Alkaloid Sequestering

GC/MS of skin extracts (SM7) indicate that *E. anthonyi* fed a control diet do not have alkaloids, but sequester dietary provided epibatidine, supporting a dietary origin of epibatidine in naturally occurring members of this species; *D. auratus* sequestered both epibatidine and sparteine, but its congener *D. tinctorius* did not sequester epibatidine; *A. femoralis* was unexpectedly able to sequester sparteine; wild-caught *D. leucomelas* contained many different alkaloids, most of which were of the pumiliotoxin class.

## 4. Discussion

Here, we will discuss our results in the context of conserved pathways for alkaloid processing in dendrobatids, considering the limitations of our experimental design. Although initially conceived as a study on alkaloid sequestering by the skin, our interpretations of the data are hindered because we could not validate the sequestration of epibatidine in our main study species, *D. tinctorius*. Furthermore, our results may to a large degree reflect an early physiological response by the frogs, because we used samples taken shortly after alkaloid (or control) consumption. We would also like to point out that the small sample size and use of alkaloids that do not occur naturally in the species included in our study might limit some of our conclusions, and hence our results and interpretations should be rather taken as testable hypotheses. Despite these partial deviations from our initial goals, our results allowed for some preliminary, yet novel insights into transcriptomic responses of poison frogs upon alkaloid uptake.

### 4.1. Gene Expression Patterns

In *D. tinctorius*, more genes were differentially expressed after the first-dose of alkaloids (single-dose treatment) than after eight days of sustained alkaloid consumption (multi-dose treatment), when compared to the controls. Both comparisons had considerable overlap in up- and down-regulated genes, and therefore we only found a few genes differentially expressed between these treatments (single-dose vs multi-dose comparison). These results are consistent with our initial hypothesis that the expression of genes related to alkaloid processing would be regulated after continued alkaloid consumption. Most genes differentially expressed in these two comparisons code for proteins that interact with each other, and we could identify enriched biological process GO-terms broadly related to muscle processes, CYPs, immune response, mitochondria, and cornification. Two of these GO-terms, immune response and cornification, were enriched when pooling together single-dose and multi-dose (i.e., alkaloid treatments) and comparing to the controls. The results of this last comparison might be more reliable because it has a higher sample size (alkaloid *N* = 5 vs control *N* = 3); however, it would be necessary to carry out more studies to confirm these are processes differentially regulated by naturally occurring alkaloids as well.

Across the species sampled in our study, we observed some concordances in gene expression patterns with the results found in *D. tinctorius*, including species that experimentally sequestered alkaloids (*D. auratus*, *A. femoralis*) and in our wild-caught frogs (*D. leucomelas*). This suggests that some general mechanisms of alkaloid processing might exist across dendrobatids. Concordant genes were included in enriched processes identified in *D. tinctorius*, such as muscle processes, immune response, mitochondria, and cornification, suggesting that these biological processes are relevant to early response (immediately and after 8 days) of poison frogs to alkaloid consumption.

### 4.2. Potential Biological Significance

The species sampled in our study did not have mutations known to confer resistance. Although we did not observe outward signs of intoxication in individuals that were fed for over a month on an epibatidine diet, there was an upregulation of genes related to muscle processes, and epibatidine is known to cause muscle contraction [35]. Poison frog alkaloids, in general, are also known to increase or decrease respiration rate in vertebrates [62]), and here we observed an upregulation of mitochondrial genes. Together, the upregulation of muscle and mitochondrial processes may indicate that epibatidine-fed frogs were impacted by the effects of this alkaloid. Furthermore, upregulation of mitochondrial processes in frogs that consume alkaloids might be linked to the higher active metabolic rate of dendrobatid species that sequester alkaloids [8], and support that alkaloid processing is energetically costly. Future research might address how alkaloid consumption influences the metabolic rate of poison frogs.

*D. tinctorius* did not sequester epibatidine, but also did not die from consuming this highly toxic alkaloid, which might imply that resistance was achieved through a mechanism other than target-site mutations or sequestering. We interpret the up-regulation of CYPs and immune response as an indication that fast alkaloid degradation could be a mechanism of resistance. In vertebrates, drug or toxin degradation usually begins with their biotransformation by CYPs in the liver. However, other tissues such as the skin also express CYPs and thus might be involved in the biotransformation of toxins as well [63]. One of the up-regulated CYPs in single-dosed *D. tinctorius* was an ortholog of the human CYP3A4, which is responsible for biotransforming over half of all drugs used in human medication [64].

Different parts of the innate and adaptive immune systems were upregulated in epibatidine fed *D. tinctorius*. To be recognized by cells of the immune system in the plasma, small molecules should bind to carrier proteins, which is facilitated by their biotransformation [19]. Although speculative, the up-regulation of several genes related to the adaptive immune system in *D. tinctorius* could suggest that alkaloids act as haptens, which allows for an alkaloid-carrier complex to be recognized as an antigen by immune system cells [65]. Supporting this, several genes related to adaptive immune system were upregulated in *D. tinctorius*, e.g. immunoglobulins. In this scenario, the adaptive immune system would activate the innate complement system, here evidenced, for example, by the upregulation of the complement protein C2 across the four species sampled. Finally, the alkaloid-carrier complex would be phagocytized and degraded by cells of the immune system [19], which could partly explain why epibatidine was not identified in the skin of *D. tinctorius*. Although the interaction of alkaloids through these pathways remains to be confirmed, this is not the first study to find a link between alkaloid processing and immune system. A recent study on *O. sylvatica* showed that the immune system protein complement C3 was up-regulated in plasma in wild individuals when compared to captive frogs [21]. Hence, alkaloid degradation or changes to the immune system might be especially important for the evolution of toxicity in dendrobatids (and possibly other poison frogs) as these pathways are conserved in vertebrates to avoid intoxication and fight infections [66,67,68]. Additional research will be needed to examine how the immune system is involved in alkaloid processing.

Across the four species sampled herein, we found a downregulation of keratins. Interestingly, one of the keratins found down-regulated in *D. tinctorius*, KRT24, was also found down-regulated in the skin of wild *O. sylvatica* [21]. This might result from the apoptosis of keratinocytes in the skin, a known cutaneous reaction to drugs that induce a strong immune response [69]. We hypothesize that, because respiration occurs mostly through the skin in amphibians, this reduction of the cornified layer of the epidermis might facilitate the oxygen exchange needed for the mitochondrial processes that are also upregulated in *D. tinctorius*. However, as a trade-off, it might also make frogs more vulnerable to infections, water loss, and UV radiation. This downregulation of keratins upon alkaloid consumption is further intriguing because two of the keratins differentially expressed in *D. tinctorius,* KRT17 and KRT19, have recently been hypothesized to relate to the diversity of coloration patterns in *D. auratus* [70]. This points to the intriguing possibility of a link between the two main traits of poison frog aposematism, coloration and alkaloid-based toxicity or unpalatability, which remains to be tested in future feeding experiments.

## Data Availability

Reads have been submitted to the Sequence Read Archive (Bioproject PRJNA562494).

## Supplementary Materials

The following are available online at https://www.mdpi.com/2073-4425/10/10/733/s1, Figure SM1. Bioinformatic pipeline used for the analysis of gene expression data. Table SM2.1. Poison frogs used for experiments. Table SM2.2. Quality and annotation of dendrobatid transcriptome assemblies. Table SM2.3. Partial alignment of nicotinic acetylcholine receptor ß2. Table SM3.1. Differential expressed transcripts for the comparison single-dose treatment vs control. Table SM3.3. Differential expressed transcripts for the comparison single-dose treatment vs multi-dose treatment. Table SM3.5. Enriched GO-terms identified by StringApp using as reference the Human genome. Comparison: single-dose vs control. Table SM3.6. Enriched GO-terms identified by StringApp using as reference the Human genome. Comparison: multi-dose vs control. Table SM3.7. Enriched GO-terms identified by StringApp using as reference the Human genome. Comparison: single-dose vs multi-dose. Table SM3.8. Enriched GO-terms identified by StringApp using as reference the Human genome. Comparison: alkaloid vs control. Figure SM4.1. Protein-protein interaction networks of proteins predicted from the differentially expressed genes in *D. tinctorius* for the comparison single-dose vs control. Figure SM4.2. Protein-protein interaction networks of proteins predicted from the differentially expressed genes in *D. tinctorius* for the comparison multi-dose vs control. Figure SM4.3. Protein-protein interaction networks of proteins predicted from the differentially expressed genes in *D. tinctorius* for the comparison single-dose vs multi-dose. Figure SM4.4. Protein-protein interaction network of proteins predicted from the differentially expressed genes in *D. tinctorius* for the comparison alkaloid vs control. Table SM5.1. LogFC for each pair of treatment-control samples. Table SM5.2. Enriched GO-terms for genes showing a consistent pattern of expression across species. Identified by StringApp using as reference the Human genome. Figure SM6.1. Heatmap of ortholog groups showing similar expression patterns (logFC of treatment-control pairs) across species and treatments. Figure SM6.2. Heatmap of ortholog groups showing similar expression patterns (logFC of treatment-control pairs) across species and treatments. Figure SM7. GC/MS chromatogram of the skin extract of *Dendrobates auratus* fed with epibatidine and sparteine.
