# Supplementary material for: Transcriptomic Signatures of Experimental Alkaloid Consumption in a Poison Frog
Source: Genes (Basel). 2019 Sep 21;10(10):733. doi: 10.3390/genes10100733 (PMC6827147; doi:10.3390/genes10100733)
Supplement: Supplementary file 1 [file genes-10-00733-s001.zip › Suppmaterials/SM1.pdf]

SM1. Sanchez et al.

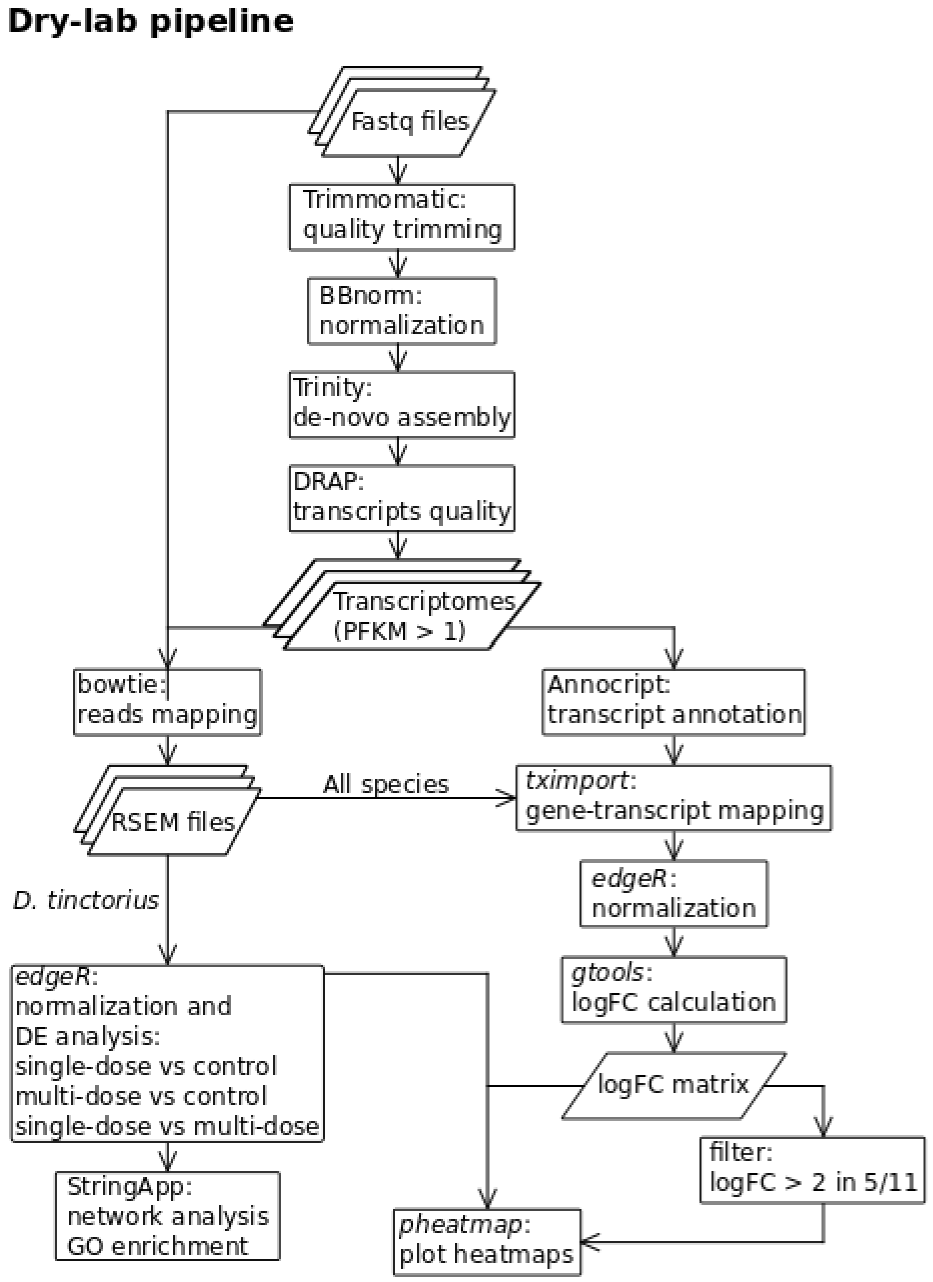

Figure SM1. Bioinformatic pipeline used for the analysis of gene expression data.
