# Supplementary material for: Transcriptomic Signatures of Experimental Alkaloid Consumption in a Poison Frog
Source: Genes (Basel). 2019 Sep 21;10(10):733. doi: 10.3390/genes10100733 (PMC6827147; doi:10.3390/genes10100733)
Supplement: Supplementary file 1 [file genes-10-00733-s001.zip › Suppmaterials/SM4.pdf]

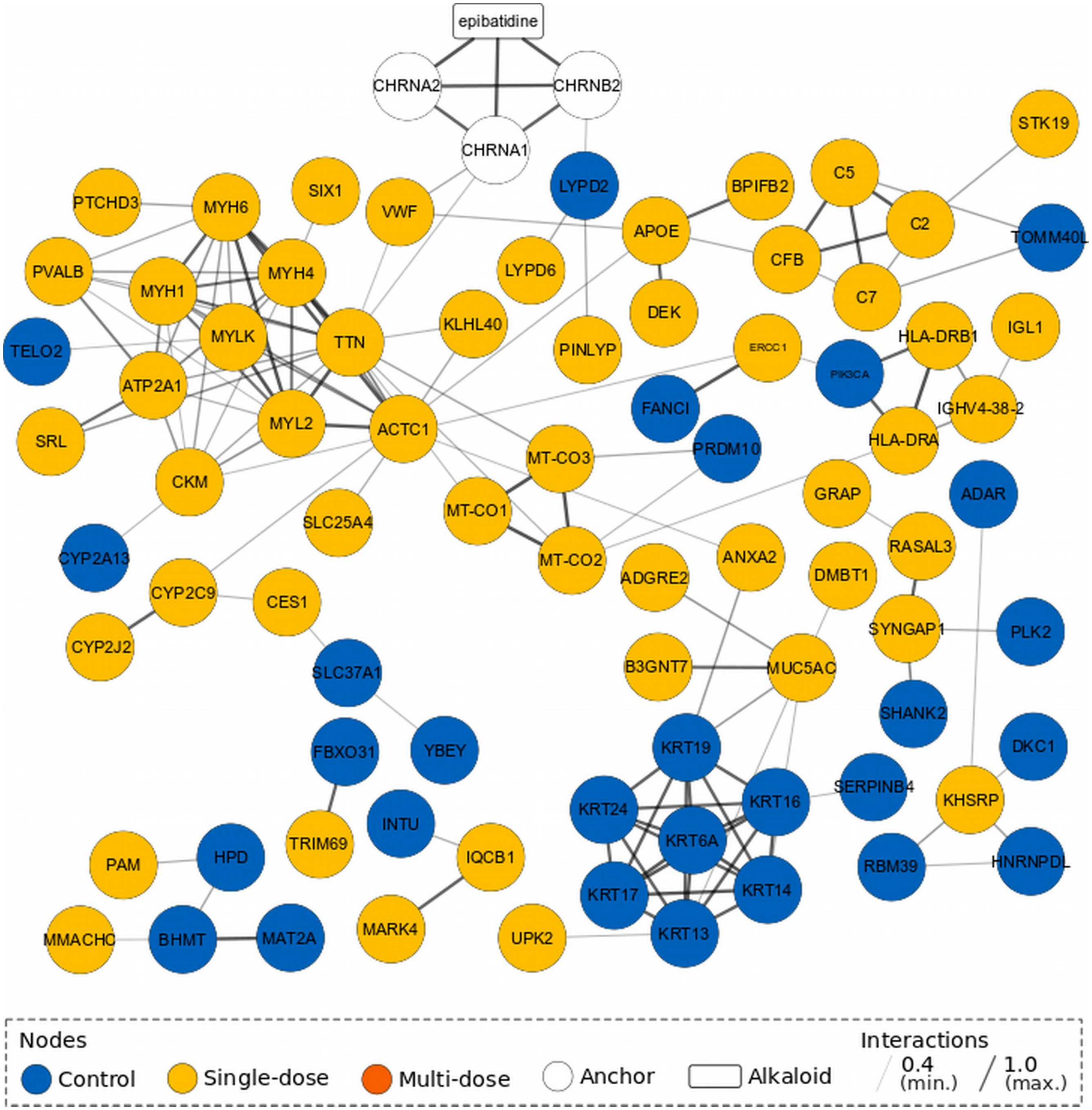

Figure SM4.1. Protein-protein interaction networks of proteins predicted from the differentially expressed genes in *D. tinctorius* for the comparison single-dose vs control. For visualization, we further connected the generated networks to epibatidine because this was the alkaloid used in both single-dose and multi-dose treatments. A network of epibatidine and nicotinic acetylcholine receptors (epibatidine's main target) was obtained from the STITCH database in the StringApp.

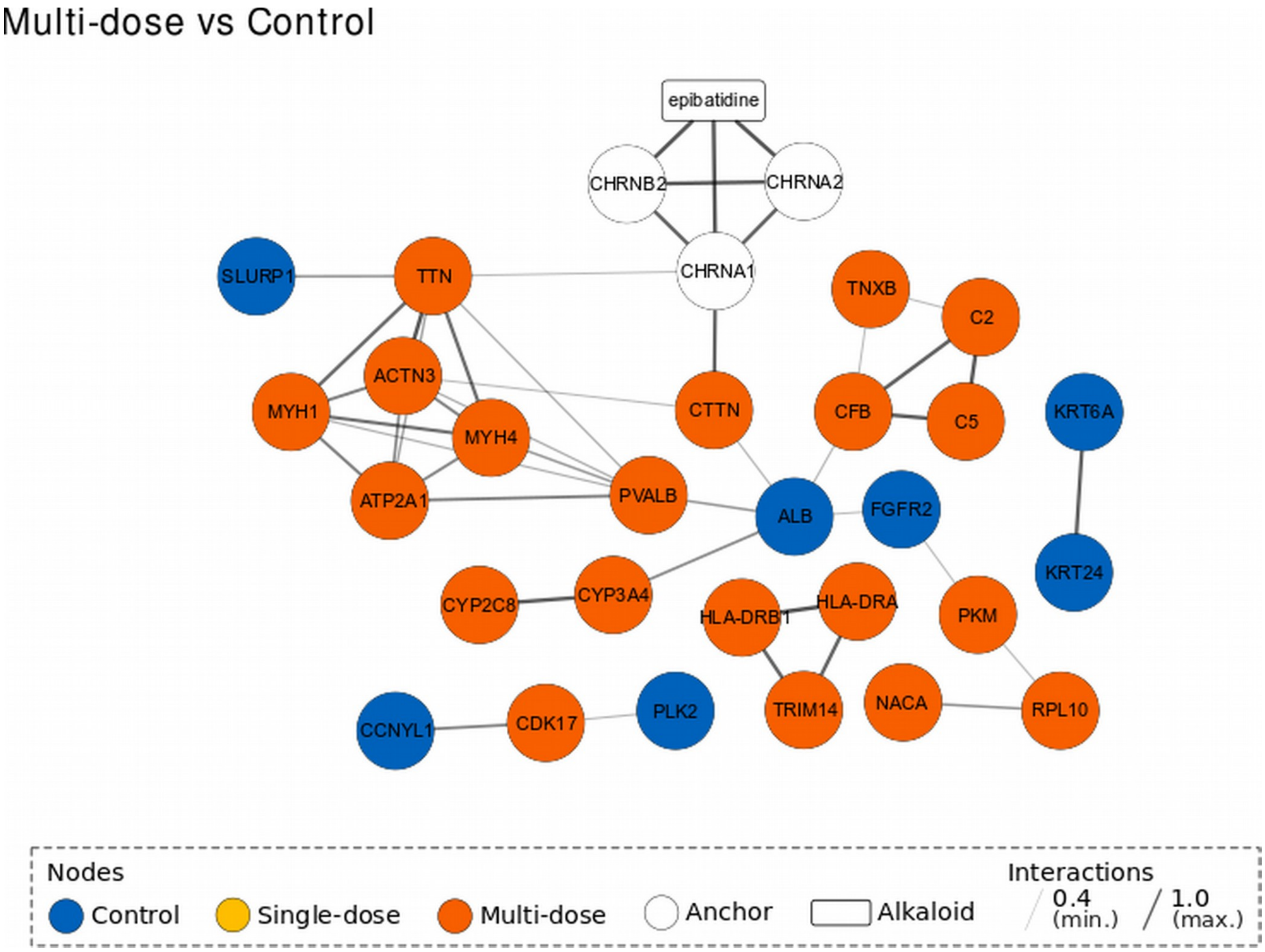

Figure SM4.2. Protein-protein interaction networks of proteins predicted from the differentially expressed genes in *D. tinctorius* for the comparison multi-dose vs control. For visualization, we further connected the generated networks to epibatidine because this was the alkaloid used in both single-dose and multi-dose treatments. A network of epibatidine and nicotinic acetylcholine receptors (epibatidine's main target) was obtained from the STITCH database in the StringApp.

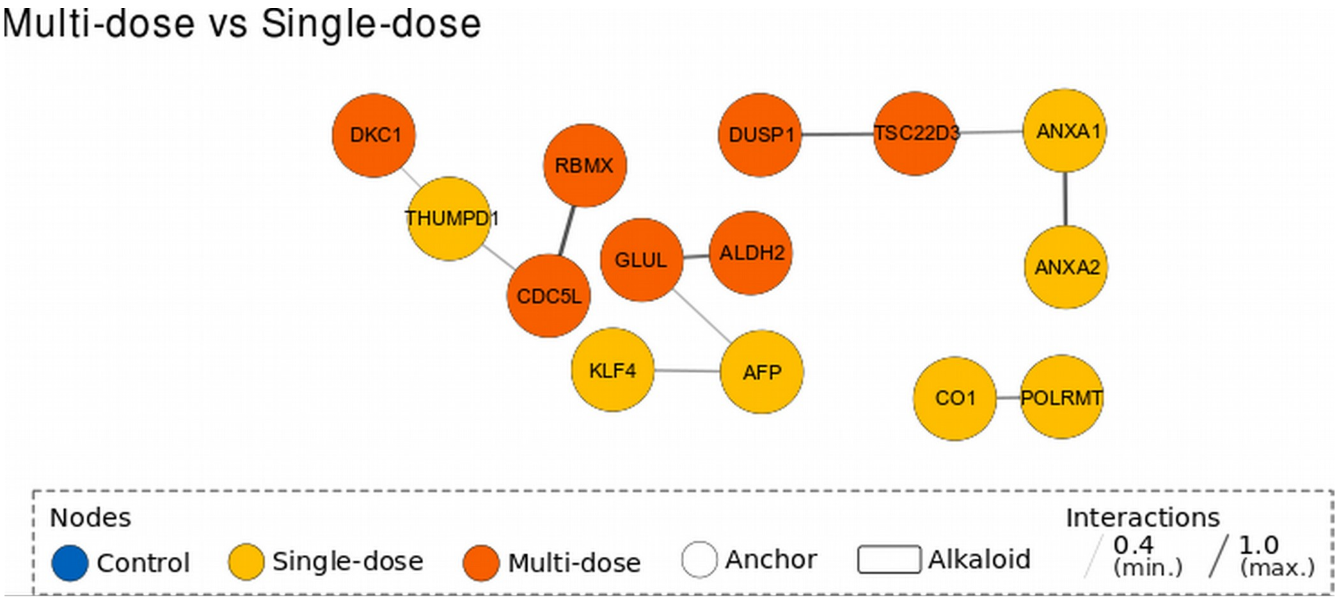

Figure SM4.3. Protein-protein interaction networks of proteins predicted from the differentially expressed genes in *D. tinctorius* for the comparison single-dose vs multi-dose.

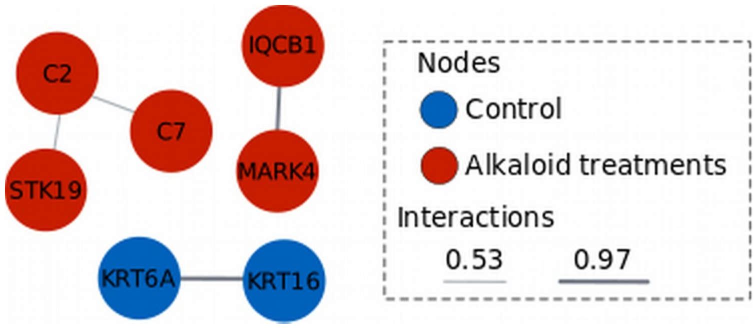

Figure SM4.3. Protein-protein interaction network of proteins predicted from the differentially expressed genes in *D. tinctorius* for the comparison alkaloid vs control.
