# Supplementary material for: Transcriptomic Signatures of Experimental Alkaloid Consumption in a Poison Frog
Source: Genes (Basel). 2019 Sep 21;10(10):733. doi: 10.3390/genes10100733 (PMC6827147; doi:10.3390/genes10100733)
Supplement: Supplementary file 1 [file genes-10-00733-s001.zip › Suppmaterials/SM6.pdf]

SM6. Sanchez et al.

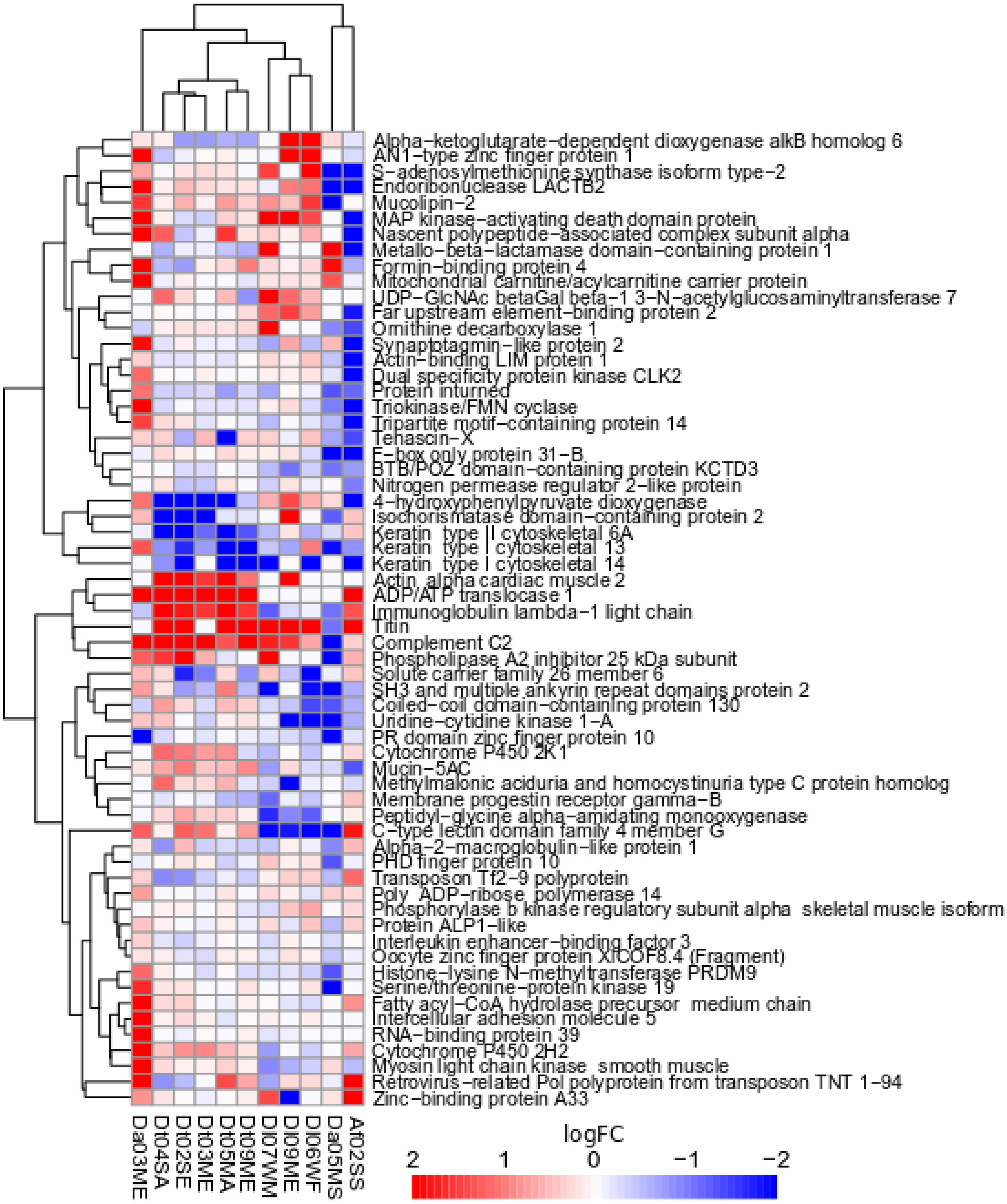

Figure SM6.1. LogFC of genes identified as differentially expressed for the comparisons single-dose vs control and multi-dose vs control in *D. tinctorius*. In the sample names, the first two characters indicate the species, the second two the individual, and the last two the treatment. Species: Dt = *D. tinctorius*, Da = *D. auratus*, Dl = *D. leucomelas*, Af = *A. femoralis*. Treatments: SE = single-dose epibatidine, SA = single dose alkaloid mixtures, ME = multi-dose epibatidine, MA = multi-dose alkaloid mixture, MS = multi-dose sparteine, WM/WF = wild. For visualization, this heatmap has a ceiling of logFC ± 2. LogFC values are given in SM5.

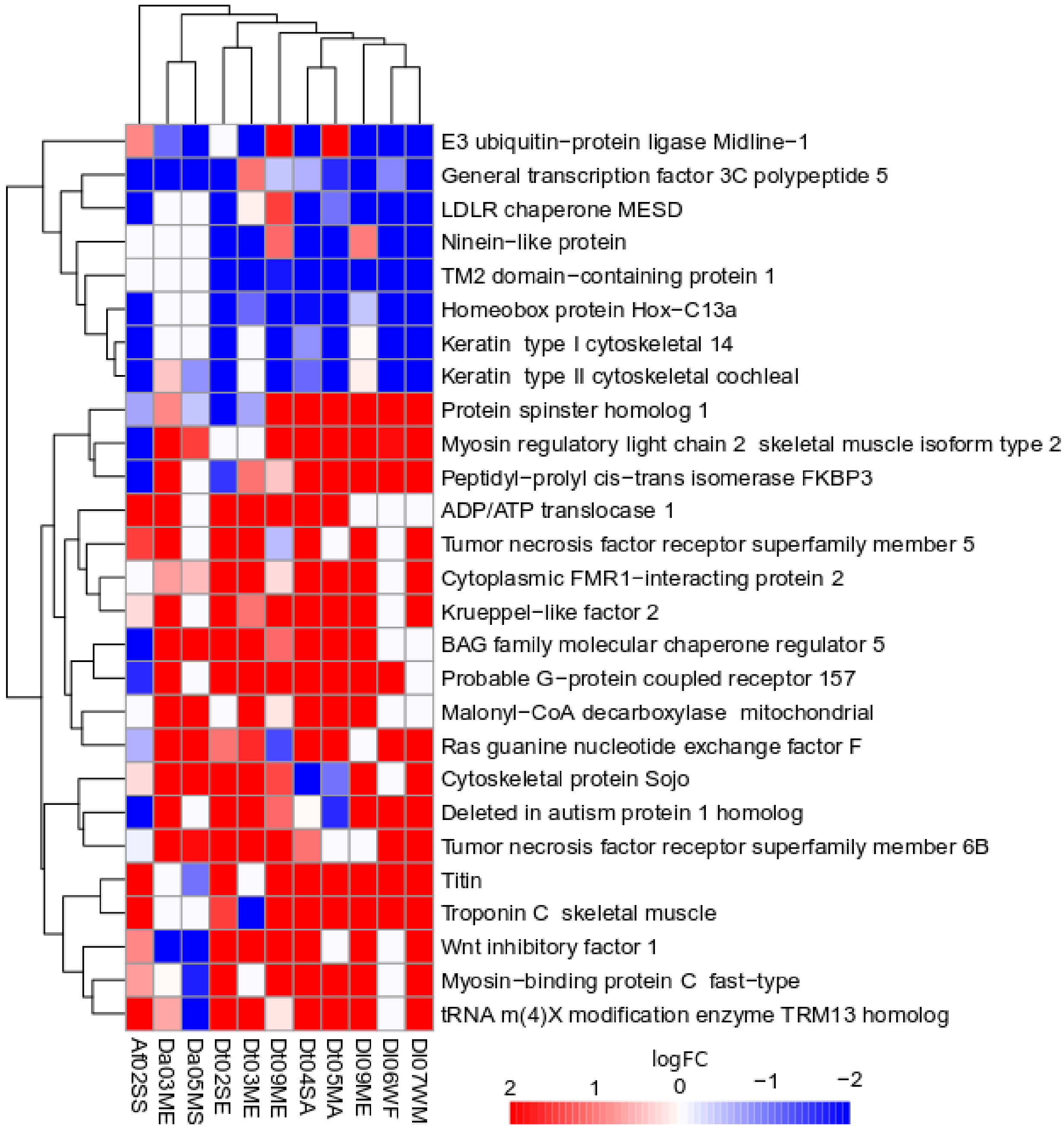

Figure SM6.2. LogFC of genes identified having a similar expression pattern across species by filtering genes with an absolute logFC > 2 for 5/11 treatment-control pairs. In the sample names, the first two characters indicate the species, the second two the individual, and the last two the treatment. Species: Dt = *D. tinctorius*, Da = *D. auratus*, Dl = *D. leucomelas,* Af = *A. femoralis.* Treatments: SE = single-dose epibatidine, SA = single dose alkaloid mixtures, ME = multi-dose epibatidine, MA = multi-dose alkaloid mixture, MS = multi-dose sparteine, WM/WF = wild. For visualization, this heatmap has a ceiling of logFC ± 2. LogFC values are given in SM5.
